# Hemiagenesis of the thyroid gland detected by coincidence—what is the clinical relevance? Case report and review of the literature

**DOI:** 10.1007/s10354-020-00783-w

**Published:** 2020-10-07

**Authors:** Peter Mikosch, Verena Weixlbaumer, Michael Irrgang, Adrian Aistleitner, Eva Trifina-Mikosch

**Affiliations:** 1 Department of Internal Medicine 2, General Hospital Mistelbach-Gänserndorf, Mistelbach, Austria; 2 University Teaching Unit, Medizinische Universität Wien/Medical University of Vienna, Vienna, Austria

**Keywords:** Thyroid development, Clinical impact of thyroid dysgenesis, Hypothyroidism, Scintigraphy, Sonography, Fehlentwicklungen der Schilddrüsenanlage, Klinische Relevanz von Fehlentwicklungen der Schilddrüse, Hypothyreose, Sonographie, Szintigraphie

## Abstract

Hemiagenesis of the thyroid gland (THA) represents a rare congenital anomaly. It is characterized by the absence of one thyroid lobe, and sometimes the isthmus as well. It can occur with all kinds of other thyroid pathologies that may be present in the remaining thyroid lobe. A case of a 21-year-old male patient is presented; he sought a thyroid consultation because of hair loss, fatigue, and problems concentrating, thus raising the suspicion of hypothyroidism. Thyroid function was normal, but sonography of the thyroid gland revealed THA of the left lobe and the isthmus. The current knowledge concerning the genesis and the clinical consequences of THA are discussed based on the current literature.

## Abstract (de)

Die Hemiagenesie der Schilddrüse (THA) stellt eine seltene Anlageanomalie dar und ist durch das Fehlen eines Schilddrüsenlappens, manchmal auch des Isthmus, charakterisiert. Die Veränderung kann zusammen mit allen anderen Erkrankungen der Schilddrüse vorkommen. Es wird der Fall eines 21-jährigen männlichen Patienten vorgestellt, der wegen Haarausfall, Müdigkeit und Konzentrationsschwierigkeiten eine Schilddrüsensprechstunde aufsuchte. Es bestand der Verdacht auf eine Schilddrüsenunterfunktion. Die Schilddrüsenfunktion war normal, aber die Sonographie der Schilddrüse ergab ein THA des linken Lappens und Isthmus. Aktuelle Erkenntnisse zur Genese und mögliche klinische Konsequenzen einer THA werden auf der Basis der Literatur zur THA diskutiert.

## Introduction

Thyroid hemiagenesis (THA) represents a rare congenital thyroid disorder that is characterized by the absence of one thyroid lobe, and sometimes the isthmus as well. Thyroid hemiagenesis can occur with all kinds of other thyroid pathologies [1–3] that may be present in the remaining thyroid lobe. The clinical symptoms of these additional thyroid pathologies, such as growing nodules (benign thyroid nodules, thyroid carcinoma), thyroid inflammation (de Quervain thyroiditis), hyperthyroidism (thyroid adenomas, Graves disease), or hypothyroidism (Hashimoto’s thyroiditis), usually lead the patient to a clinical investigation [4]. Ectopic sublingual thyroid gland [5–7] and thyroglossal duct cysts [8] have been reported in combination with THA as well. Pathologies of the parathyroid gland or other pathologies within the neck region with growing masses can also lead to a thyroid investigation and detection of THA [9–11]. Thus, THA is detected in most reported cases only by coincidence, and the majority of cases with THA therefore likely remain undetected, as THA by itself frequently causes no clinical symptoms. However, the question remains whether THA really causes no symptoms or clinical consequences.

Based on a case with a coincidentally discovered THA but without any other thyroid pathologies, the patient raised some questions concerning THA: 1) Does the thyroid gland reduced to only one lobe lead to hypothyroidism and/or 2) a compensatory lobe enlargement of the remaining thyroid lobe? 3) Are patients with THA susceptible to any other thyroid disorders?

To answer these questions, the current literature on THA using the term “thyroid hemiagenesis” was searched in PubMed. In particular, publications dealing with cases of THA without other accompanying thyroid disorders were selected in order to evaluate the consequences of THA on its own.

## Case presentation

A 21-year-old man attended an endocrinological consultation with the suspicion of hypothyroidism due to ongoing fatigue, increased hair loss, and problems concentrating.

**Fig. 1:**
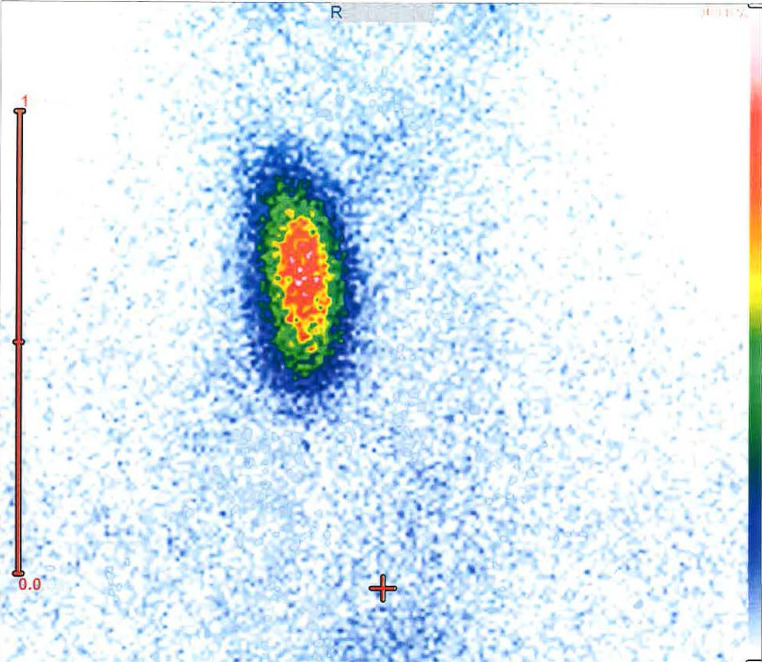
^99m^Tc-pertechnetate scintigraphy: homogeneous tracer accumulation over the right thyroid lobe. There is no tracer accumulation within the left side of the thyroid bed, thus proving the diagnosis of thyroid hemiagenesis. Furthermore, no ectopic tracer accumulation is seen, thus excluding other ectopic thyroid tissue

The clinical examination of the patient revealed a normal physical status. Palpation of the thyroid region was unobtrusive. Laboratory testing showed a normal thyroid function with a TSH of 1.98 mU/l (normal range 0.27–4.2 mU/l), fT4 1.3 ng/dl (normal range 0.93–1.7 ng/dl), and fT3 3.8 pg/ml (normal range 2.0–4.4 pg/ml). The thyroid antibodies TAK, TPO, and TRAK were all negative. The iodine content in the serum was slightly reduced at 43 µg/l (normal range 50–90 µg/l), consistent with a mild iodine deficiency. All other laboratory parameters were normal except for 25-hydroxy vitamin D3, which was decreased at 17.7 ng/ml, resembling vitamin D deficiency. Remarkably, sonography of the thyroid gland showed a right lobe of normal size (41 × 17 × 14 mm) with a normal homogeneous echo pattern, but no left thyroid lobe and no isthmus. ^99m^Tc-pertechnetate scintigraphy showed a homogeneous and normal tracer accumulation of the right lobe but missing tracer accumulation within the left side and isthmus, thus confirming thyroid hemiagenesis (Fig. 1).

Because of the iodine deficiency, the patient was advised to increase his iodine intake by iodine-rich food. No medical treatment was established.

Four years later the patient presented himself without any complaints. Sonography showed an unchanged size of the right lobe (43 × 16 × 15 mm) and a homogeneous echo pattern with no thyroid nodules. The TSH level was still normal at 1.44 mU/l.

Discussing the results of the investigations with the patient, he raised the question of what impact THA would have for him in the short term and long term.

## Epidemiology of thyroid hemiagenesis

Thyroid hemiagenesis is a rare congenital anomaly of the thyroid gland. In an extensive literature search we found a total of 256 reported cases in 1999 [2]. Since then, almost 500 additional cases have been reported, bringing the total number of published cases of THA to almost 800 in the current literature. It shows a female predominance, with a female-to-male ratio between 1.3 : 1.00 [2], 1.4 : 1.00 [4], and 1.67 : 1.00 [12]. Absence of the left lobe is more frequently seen, with a left-to-right ratio of 3.6 : 1 [2, 3], and the isthmus being present in 44% [2].

Surveys in children and adults showed a THA prevalence of 0.016%–0.5% in children and 0.022%–0.25% in adults (Table 1; [1, 2, 12–20]). However, when children with congenital hypothyroidism were studied with sonography for the underlying pathology, a prevalence of 2.44%–3.7% was found in two surveys [21, 22]. In this respect, THA has to be regarded as a relevant cause for congenital hypothyroidism, and sonography in these children should be performed as early as possible to uncover THA or other abnormalities of thyroid development.

**Table 1:** Overview of thyroid hemiagenesis (THA) prevalence based on studies using sonography to diagnose THA. The different settings were based either on preselection by congenital hypothyroidism, field studies in children and young adults, or results acquired retrospectively in thyroid outpatient wards

Author (Ref.)	*n* screened	Setting of sonography	*n* THA	Comments	THA prevalence (%)
Borges [21]	41	Congenital hypothyroidism	1	All patients on L‑thyroxine therapy at time of investigation	2.44
Hashemipour [22]	437	Congenital hypothyroidism	NA	All hypothyroid	3.7
Duarte [19]	964	Field study, children	NA	–	0.5
Shabana [17]	2845	Field study, children	6	–	0.2
Korpal-Szczyrska [15]	4004	Field study, children	2	Both euthyroid	0.05
Hayashida [20]	4365	Field study, children	2	–	0.05
Maiorana [16]	24,032	Field study, children	12	9 tested for thyroid function, all euthyroid, but TSH higher than in controls	0.05
Gursoy [18]	4772/2935	Field study, children/adults	2	All euthyroid	0,025
Suzuki [12]	299,908	Field study, children and young adults	67	In THA, contralateral thyroid lobe larger compared with normal thyroids	0.016 (male)
					0.027 (female)
Schneider [1]	NA	Thyroid ward, adults	13	Associated additional thyroid disorders in all patients	0.08
Gursoy [18]	4833	Thyroid ward	12	All euthyroid, 3 cases with no additional thyroid disease	0.25
Berker [14]	6242	Thyroid ward, adults	10	2 with no additional thyroid disease, 9 patients clinically asymptomatic	0.16
Mikosch [2]	71,500	Thyroid ward, adults	16	1 hyperthyroid, 7 hypothyroid (4 with iodine deficiency), 9 euthyroid	0.022

*NA* not available

## Embryological development of the thyroid gland and genetic causes of thyroid hemiagenesis

The exact causes of the disturbed thyroid development leading to THA are still not fully understood. The embryological development of the thyroid starts with a median endodermal diverticulum on the floor of the pharynx at the foramen cecum, which then elongates and forms a bilobed diverticulum. It later descends in the neck anterior to the trachea to its final position. In THA the formation of the bilobed diverticulum is disturbed, leading to the development of only one lobe [23].

A genetic background has been suspected for years, as THA has been observed in monozygotic twins [24, 25], among sisters [26], and within a family [25, 27]. The morphogenetic process of thyroid gland development is regulated by both cell-autonomous (e.g., activated by NKX2‑1, FOXE1, PAX8, HHEX) and mesoderm-derived (e.g., mediated by TBx1 and fibroblast growth factors) mechanisms in concert to promote growth and survival of progenitor cells [28]. Fagman et al. [29] showed that the T‑box transcription factor TBx1 has great influence on the development of the thyroid gland. In TBx1 −/− mice, the downward translocation of Titf1/Nkx2.1-expressing thyroid progenitor cells is delayed. During further development the thyroid fails to form symmetric lobes, and the hypoplastic gland frequently has a unilateral position resembling THA. These findings indicate that Tbx1 regulates intermediate steps of thyroid development by a non–cell-autonomous mechanism [29].

Szczepanek et al. [30] found aside a high polymorphic variability of FOXE1-polyAla that shorter variants of FOXE1-polyAla were underrepresented in subjects with familial THA. FOXE1-polyAla tract expansion may thus contribute to familial, but not sporadic, forms of THA [30]. Cerqueira et al. [31] performed a mutation screening in different genes (PAX‑8, NKX2‑5, TSH‑R, HES-1) that are involved in thyroid development. In this study, no mutations were detected in any of the candidate genes [31]. Castanet et al. [32] screened a cohort of 22 patients with thyroid dysgenesis including THA and also found no PAX‑8 mutations. However, Szczepanek-Parulska et al. [33] described an alternative 3’acceptor site in exon2 of the PAX‑8 gene, which might be associated with THA. Budny et al. [34] analyzed a cohort of 34 sporadic patients diagnosed with THA and a three-generation family by comprehensive genomic examination. Like other investigators screening the transcription factors directly involved in the thyroid development, they found no causative mutations. However, genomic examination showed four recurrent defects sporadically affecting highly conservative proteasome genes (PSMA1, PSMA3, PSMD3). In addition, in the thyroid hemiagenesis family, a splice-site mutation (c.612T > C cDNA.1170T > C, g.3271T > C) in the proteasome gene PSMD2 was found in both the mother and the daughter who were affected by THA. Kizys et al. [35] reported on polymorphisms in the HOXB3, HOXD3, and PITX2 genes in familial and sporadic cases of THA. Kim et al. [36] presented one case of THA and congenital hypothyroidism with 22q11.2 microduplication, suggesting that 22q11.2 may also be considered as a possible cause of THA. The frequent coexistence of cardiac anomalies suggests that the thyroid morphogenetic process may also depend on proper cardiovascular development [29]. However, in the majority of patients with THA, the genetic background still remains unknown [37]. Thus, according to the current knowledge, THA is likely a polygenetic disorder. In addition, epigenetic factors may also be involved in the pathogenesis of THA [31, 35].

## Clinical consequences of thyroid hemiagenesis

Thyroid hemiagenesis can occur with all kinds of thyroid pathologies [1, 2] that may be present in the remaining thyroid lobe. The symptoms of these additional thyroid pathologies, such as growing nodules, thyroid inflammation, hyperthyroidism or hypothyroidism, lead the patient to a clinical investigation [4]. Many patients with THA present without any symptoms, and THA may be found only by coincidence. However, questions arise regarding which clinical consequences may be due to THA itself and not to additional thyroid pathologies that cause symptoms and lead to diagnosis.

Suzuki et al. [12] conducted a large survey in 299,908 children and young adults from Japan. In comparison to subjects with normally formed thyroid glands, persons affected by THA revealed a larger contralateral thyroid lobe. According to the authors, this may have been caused by a compensatory feedback mechanism to prevent hypothyroidism [12]. Ruchala et al. [38] also found in their cohort study that patients with THA had higher TSH levels than the controls with normal thyroid glands. This observation seems plausible, as the organ volume for thyroid hormone production will be smaller in a unilobulated thyroid gland. In this context it is thus of interest to what extent hypothyroidism is present in early life. In several studies analyzing the causes of congenital hypothyroidism [12, 16, 17, 21, 22, 39–42], all studies showed that THA can be a cause of congenital hypothyroidism. Maiorana et al. [16] found in their survey that children with thyroid hemiagenesis had an average serum TSH significantly higher than that of 18 matched controls (2.8 ± 0.6 mU/l vs. 1.9 ± 0.5 mU/l; *P* < 0.001), and Cerqueira et al. [31] reported that THA was the cause of congenital hypothyroidism in 6% of the cases studied. Two other surveys that screened patients with congenital hypothyroidism found THA with a prevalence of 2.33%–3.7% as the cause of congenital hypothyroidism [21, 22].

Putting these results together, due to the reduced organ volume, THA seems to be associated with increased TSH levels [16, 38], sometimes even causing hypothyroidism. Furthermore, the increased TSH levels are a growth stimulus for the thyroid cells, leading to thyroid hypertrophy and finally to compensatory enlargement of the present thyroid lobe. Over the long term with constantly increased TSH levels, the development of thyroid nodules may be promoted. Interestingly, Szczepanek-Parulska et al. [43] reported in their study group a higher prevalence of thyroid antibodies in patients with THA compared with individuals with normal thyroid glands, thus suggesting evidence that THA may also promote the development of immune thyroid disorders.

In clinical terms, the question arises as to how patients with THA should be followed up. Based on the current literature, no answers to this question exist so far. Two aspects have to be considered when managing patients with THA: first, management and follow-up of THA itself, and second, diagnosis, management, and follow-up of thyroid disorders in the remaining thyroid lobe.

Ultrasonography is the method of choice to diagnose THA [2, 14, 21, 22, 39, 44, 45] and to detect any morphological abnormalities within the remaining lobe. Furthermore, ultrasonography can be used to monitor patients with THA and associated thyroid disorders such as thyroid nodules concerning nodule growth. Scintigraphy with ^99m^Tc-pertechnetate or ^123^I can give proof of THA (Fig. 1) and is helpful for detecting ectopic thyroid tissue [2, 45, 46]. After diagnosis of THA, scintigraphy can also be used to diagnose thyroid pathologies in the remaining lobe associated with hyperthyroidism (thyroid adenoma, Graves disease) or growing thyroid nodules with suspicion of malignancy [2, 46]. Fine-needle aspiration biopsy can be of help in determining the character of suspicious thyroid nodules [2]. Because patients with THA seem to be prone to hypothyroidism, regular control of thyroid hormones, in particular TSH, seems to be warranted to detect any thyroid dysfunction at an early stage. Due to the higher reported rate of autoimmune disorders in THA [43], a consequent evaluation of thyroid antibodies seems to be warranted when THA has been diagnosed.

Because patients with hemiagenesis may have a higher tendency toward development of hypothyroidism due to the reduced organ volume, and because iodine deficiency can also be a cause of hypothyroidism, it seems to be advisable to analyze the iodine content in the urine or blood, in particular in iodine-deficient areas. Mikosch et al. [2] found 16 cases of THA in their analysis of 71,500 cases over 9 years within an endemic goiter area. Seven of these 16 patients with THA were hypothyroid, and four of them revealed iodine deficiency as diagnosed by low iodine excretion in the urine (the remaining three patients were not tested for iodine excretion in urine). The patient presented in this paper also most likely had a suboptimal iodine supply, based on low iodine levels in the serum. These findings point out that evaluation of iodine status, particularly in iodine-deficient areas, is of importance in patients with THA in order to rule out iodine deficiency as a possible cause of hypothyroidism and/or cause of goiter development. In the case of hypothyroidism, this will enable the clinician to treat it with either L‑thyroxine supplementation or, in cases of iodine deficiency, with iodine supplementation alone or in combination with L‑thyroxine.

## Conclusion

For patients with THA and a normal thyroid function, but with no other thyroid pathologies, there are no relevant clinical consequences in the short term. However, based on the current literature, a susceptibility to other thyroid pathologies—especially hypothyroidism, increased rates of thyroid autoimmune diseases, and thyroid enlargement (diffuse or nodular)—may be possible consequences in the long term. Thus, regular follow-up visits concerning thyroid function in particular seem to be advisable in these patients in order to diagnose possible hypothyroidism, immune disease, or thyroid growth at an early stage.

Patients with THA and additional thyroid pathologies should be cared for as any other patient with a normally formed thyroid gland, including laboratory testing, sonography, scintigraphy, and fine-needle aspiration biopsy [2].
